# Location of Intra- and Extracellular *M. tuberculosis* Populations in Lungs of Mice and Guinea Pigs during Disease Progression and after Drug Treatment

**DOI:** 10.1371/journal.pone.0017550

**Published:** 2011-03-21

**Authors:** Donald R. Hoff, Gavin J. Ryan, Emily R. Driver, Cornelius C. Ssemakulu, Mary A. De Groote, Randall J. Basaraba, Anne J. Lenaerts

**Affiliations:** 1 Department of Microbiology, Immunology, and Pathology, Colorado State University, Fort Collins, Colorado, United States of America; 2 Council for Scientific and Industrial Research (CSIR), CSIR Biosciences, Pretoria, South Africa; Institut Pasteur, France

## Abstract

The lengthy treatment regimen for tuberculosis is necessary to eradicate a small sub-population of *M. tuberculosis* that persists in certain host locations under drug pressure. Limited information is available on persisting bacilli and their location within the lung during disease progression and after drug treatment. Here we provide a comprehensive histopathological and microscopic evaluation to elucidate the location of bacterial populations in animal models for TB drug development.

To detect bacilli in tissues, a new combination staining method was optimized using auramine O and rhodamine B for staining acid-fast bacilli, hematoxylin QS for staining tissue and DAPI for staining nuclei. Bacillary location was studied in three animal models used in-house for TB drug evaluations: C57BL/6 mice, immunocompromised GKO mice and guinea pigs. In both mouse models, the bacilli were found primarily intracellularly in inflammatory lesions at most stages of disease, except for late stage GKO mice, which showed significant necrosis and extracellular bacilli after 25 days of infection. This is also the time when hypoxia was initially visualized in GKO mice by 2-piminidazole. In guinea pigs, the majority of bacteria in lungs are extracellular organisms in necrotic lesions and only few, if any, were ever visualized in inflammatory lesions. Following drug treatment in mice a homogenous bacillary reduction across lung granulomas was observed, whereas in guinea pigs the remaining extracellular bacilli persisted in lesions with residual necrosis.

In summary, differences in pathogenesis between animal models infected with *M. tuberculosis* result in various granulomatous lesion types, which affect the location, environment and state of bacilli. The majority of *M. tuberculosis* bacilli in an advanced disease state were found to be extracellular in necrotic lesions with an acellular rim of residual necrosis. Drug development should be designed to target this bacillary population and should evaluate drug regimens in the appropriate animal models.

## Introduction

The standard regimen for tuberculosis (TB) requires 6–9 months of daily, multidrug therapy to achieve sterilization without relapse [1], [2]. The lengthy treatment regimen is thought to be necessary to eradicate a small sub-population of *M. tuberculosis* bacilli that persist in the face of drug pressure [3]. The ability of *M. tuberculosis* to adapt to a changing environment and persist in certain locations of the host despite a vigorous adaptive immune response, likely contributes to the difficulty in curing TB with antimicrobial drugs [3]–[6]. Several *in vitro* studies to date have shown that the environment surrounding *M. tuberculosis* can alter the metabolic state and replication rate of the bacilli thereby rendering them refractory to drug treatment [7]–[11]. In contrast to the controlled conditions of culture environments *in vitro*, very little information is available on the far more complicated *in vivo* conditions of the bacteria such as in human lung lesions [5]. We previously described that *M. tuberculosis* exists as multiple populations in most *in vitro* and *in vivo* conditions, even within a seemingly single microenvironment [12]. More understanding of the bacterial location in the lung throughout disease progression and the bacterial *in vivo* microenvironment is needed to elucidate the host-pathogen interaction further.

Human TB infected lungs generally show a heterogeneity of lung lesion types often with necrosis in the center and surrounded by a peripheral rim of fibrosis [13]. A better understanding of tuberculosis lesion pathogenesis is emerging from evaluating animal models that demonstrate diverse *in vivo* responses to experimental infections. Animal infection models are essential to vaccine and drug development by generating the data that are a prerequisite for clinical trials. The standard laboratory mouse models have provided critical information on the efficacy of a novel compound in TB drug development as well as on safety and pharmacokinetics [14]–[17]. The drawback of the standard mouse infection models for tuberculosis is their lack of advanced lesion types as the progression of disease rarely reaches the stages of extensive necrosis and calcification in the lungs [18]. Numerous studies have demonstrated that the standard laboratory mouse fails to show significant necrosis and thus hypoxia in lung lesions [19]–[21]. Therefore, the addition of a secondary animal model showing a broad range of pathological features might be necessary at later stages of TB drug development to assess drug activity against persisting bacteria in necrotic lesions. Guinea pigs infected with *M. tuberculosis* show greater similarities to natural infections in humans, as these animals show hypoxia, necrosis and calcification [21]–[25]. Recently, more attention has been brought to the importance of comparative pathology and pathogenesis in different animal models [21], [26], yet little information is available regarding the actual location of the bacilli in these pulmonary lesions across animal models. There are at least two reasons that may explain the lack of this information. Many animal models showing these advanced lesion types, such as the non-human primates and the current rabbit model, show only low bacterial numbers per lesion which makes visualization in tissue sections very difficult [21], [27], [28]. In addition, the staining methods used by most are labor-intensive and time consuming especially when numerous organ sections from multiple animals require microscopic evaluation.

To visualize *M. tuberculosis* either in tissues or in sputum smears, the detection method most commonly employed is the Ziehl-Neelsen (ZN) acid-fast stain [29]–[35]. Fluorescence is much preferred over brightfield microscopy because it provides greater sensitivity at a lower scanning magnification, yielding more consistent results with less user-fatigue [36]–[38]. Among the various acid-fast, fluorochrome staining reagents used in TB detection, the most common are auramine O and the auramine-rhodamine (AR) combination. A comprehensive review on the comparison of the AR and ZN staining techniques to detect *M. tuberculosis* in sputum smears demonstrated that both techniques showed similar specificity, but the AR technique was found to be more sensitive and facilitated easier microscopic evaluations versus the ZN stain [39]. One drawback of using fluorescent acid-fast stains is that tissue architecture is obscured due to the counterstain that darkens all but the bacilli. Only a few reports describe the use of AR in combination with other tissue stains on tissue samples [40]–[42]. In this study, a combination staining method was developed to not only identify *M. tuberculosis* bacilli, but to also navigate through tissue histology. This method enables bacilli to be accurately located within tissue sections. In an earlier study, we described that treatment of guinea pigs with the experimental drug TMC207 almost completely eradicated bacteria throughout the lesions, and that some remnants of acid fast bacilli remaining after treatment were observed to be extracellular, in the acellular rim of primary necrotic lesions by the Ziehl-Neelsen method [23]. In this study a thorough, in-depth comparison of the bacterial location in several animal models was performed during the progression of disease as well as after drug treatment using the newly developed combination staining method.

To date, the activity of novel compounds *in vivo* is generally measured by the reduction in colony forming units (CFU) from organ homogenates of infected animals with little knowledge relating to pathology, location of the bacilli at the start and during drug treatment, and the effect of drug activity in different locations in the lung. To address these three aspects, we studied the different standard animal models used in our laboratory for evaluation of experimental compounds against tuberculosis ; a short term *M. tuberculosis* infection model using immunocompromised IFN-γ gene knockout mice [43], the immunocompetent C57BL/6 mouse model [44] and the guinea pig model [23], [45]. All *M. tuberculosis* infections were performed by low dose aerosol infection (using an initial inoculum of 30–100 CFU in the lungs of the animals). The ultimate goals of this study were: 1) to understand the pathogenesis across the animal models in terms of lung lesion pathology and to record the location of the bacilli, 2) to gain information about drug effects on the pathology in the different animal models, and 3) to study the location of the persistent bacterial population remaining after drug treatment in the lung. A better understanding of the animal models used in TB drug development is critical in delineating the *in vivo* activity of an experimental compound against certain populations of *M. tuberculosis* bacteria based on their location. In addition, this will ensure the use of the appropriate animal model for the evaluation of new drug classes.

## Results

### Drug efficacy in immunocompetent vs. immunocompromised mice

**Figure 1:**
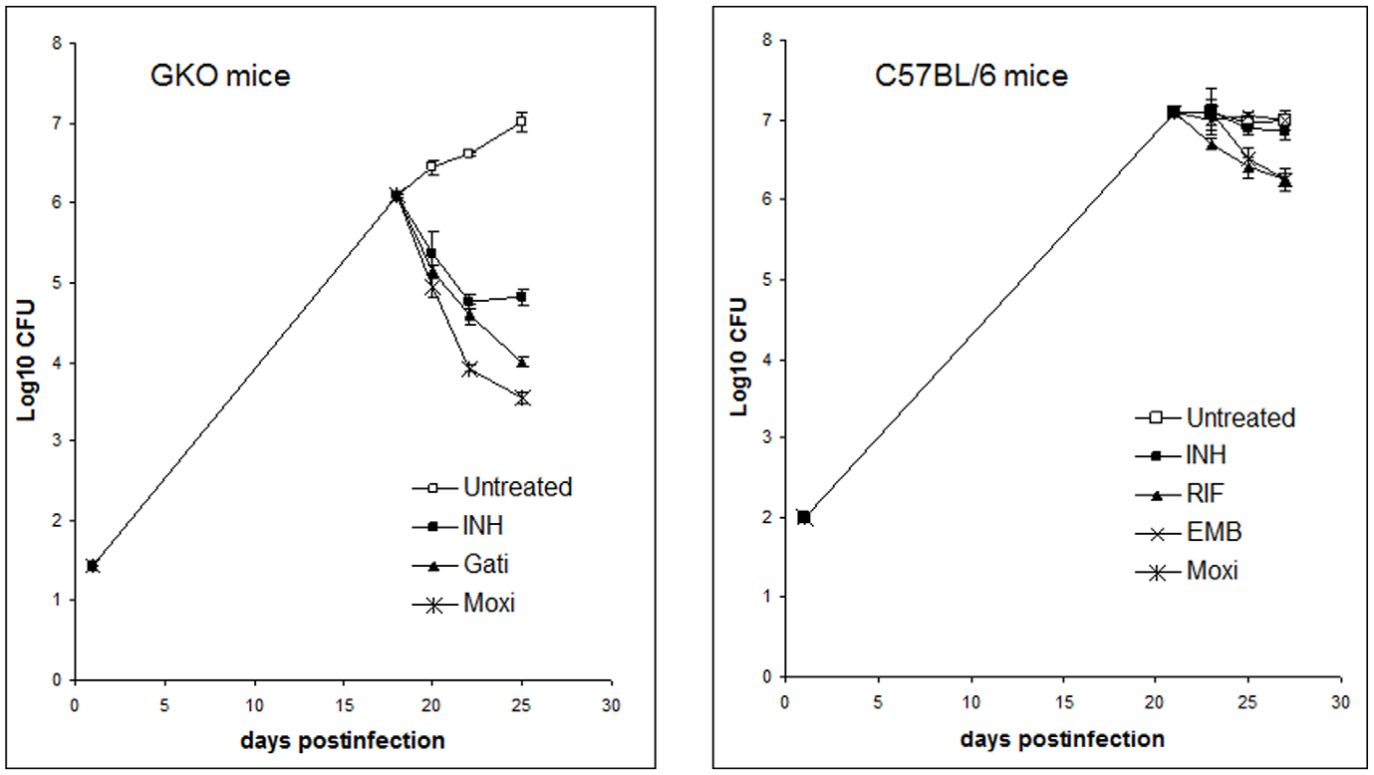
Numbers of viable *M. tuberculosis* organisms in lungs of infected IFN-γ knock-out and C57BL/6 mice after drug treatment. Mice were infected with *M. tuberculosis* strain Erdman and treated with drugs starting 18 to 21 days after aerosol infection; with isoniazid (INH), rifampin (RIF), ethambutol (EMB), gatifloxacin (Gati) or moxifloxacin (Moxi). Sacrifice times were at 2, 5 and 7 days of drug treatment. Data points represent mean log_10_ viable bacilli +/− standard error present in whole lung homogenates.

The efficacy of several clinically used TB drugs was evaluated in our standard C57BL/6 versus GKO mouse model infected via a low dose aerosol of *M. tuberculosis* (Fig. 1). At the start of treatment, the bacterial load in the lungs reached ∼7.0 log_10_CFU in C57BL6 mice (at 21 days after aerosol infection) and 6.2 log_10_CFU in GKO mice (at 18 days after aerosol). At the completion of the study (at 28 days after aerosol infection), the bacterial load in the untreated control group remained largely unchanged for C57BL/6 mice (*P>0.05*), whereas the bacillary burden in GKO increased by more than 1 log_10_CFU (Fig. 1). The activities of the tested drugs were evaluated over 7 days with sacrifice point after 2, 5 and 7 days of treatment. INH reduced the bacterial load only slightly over 7 days in the lungs of the C57BL/6 mice (0.25 log_10_CFU reduction), whereas in the immunocompromised GKO mice, INH showed significant activity mainly over the first 5 days of treatment (0.85 log_10_CFU reduction) (*P<0.05*) (Fig. 1). The difference in drug activity between the two mouse strains was even more pronounced with MXF treatment at 100 mg/kg, which reduced the bacterial load in C57BL/6 mice by 0.85 log_10_CFU and by more than 3.5 log_10_CFU in GKO mice after 7 days of treatment when compared to untreated mice at the start of treatment *(P<0.05)*.

### Intra- and extracellular stages of M. tuberculosis in lung lesions of GKO mice

**Figure 2:**
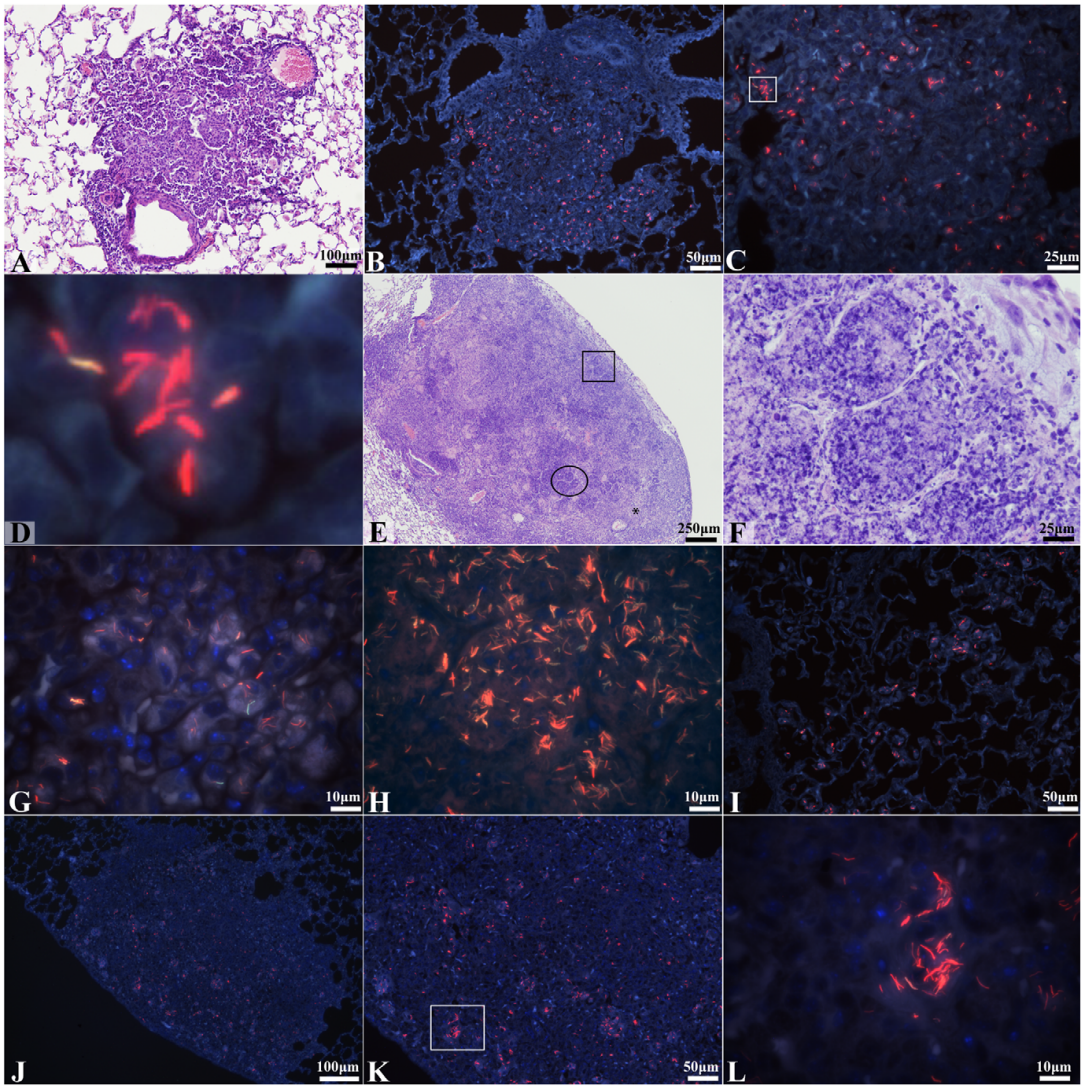
Intra-and extracellular *M. tuberculosis* bacilli in lungs throughout infection from *M. tuberculosis* infected GKO mice and after 9 days of INH drug treatment. (AR) auramine-rhodamine, hematoxylin QS and DAPI; (H&E) hematoxylin and eosin. (A) An inflammatory lesion from lungs of an *M. tuberculosis* infected GKO mouse 18 days after aerosol infection. The inflammatory lesion shows a mix of macrophages and granulocytes arranged in an unorganized manner (H&E, 100× magnification). (B) Fluorescent image of an inflammatory lesion from lungs of an *M. tuberculosis* infected GKO mouse 18 days after aerosol infection. Intracellular red fluorescent AR+ stained bacilli were predominantly found uniformly distributed throughout inflammatory lesions at this time (AR, 200× magnification). (C) High magnification fluorescent image of the inflammatory lesion in figure 2B. The lesion shows intracellular AR+ bacilli located within various macrophage cells (AR, 400× magnification). (D) Cropped image taken from figure 2C (square) showing multiple AR+ stained bacilli within a single macrophage cell (AR, digital magnification). (E) A necrotic inflammatory lesion from lungs of an *M. tuberculosis* infected GKO mouse 28 days after aerosol infection. Multiple foci of intense basophilic staining are seen in alveolar spaces as they accumulate cellular necrotic debris (H&E staining, 40× magnification). (F) High magnification of alveolar spaces filled with cellular necrotic debris located within the inflammatory lesion depicted in figure 2E (square) (H&E staining, 400× magnification). (G) High magnification of a non-necrotic area within the inflammatory lesion seen in figure 2E (star) taken from a serial tissue section. Fluorescent image shows a number of intracellular AR+ stained bacilli residing within macrophages (AR, 1000× magnification). (H) High magnification of an alveolar space filled with necrotic debris located within the inflammatory lesion shown in figure 2E (circle) taken from a serial tissue section. Image shows a high number of extracellular AR+ stained bacilli residing among cellular necrotic debris situated in alveolar spaces (AR, 1000× magnification). (I) Fluorescent image of an inflammatory lesion from lungs of an *M. tuberculosis* infected GKO mouse 28 days after aerosol infection showing intracellular AR+ stained bacilli within multiple macrophages dispersed in non-granulomatous tissue (AR, 200× magnification). (J) AR+ bacilli within a remaining inflammatory lesion from lungs of an *M. tuberculosis* infected GKO mouse after 10 days of INH treatment (AR, 100× magnification). (K) Higher magnification of the inflammatory lesion seen in figure 2J. The lesion shows that the majority of remaining AR+ stained bacilli are intracellular within macrophages (AR, 200× magnification). (L) High magnification of the inflammatory lesion seen in figure 2K (square). This fluorescent image shows AR+ bacilli were predominantly found within macrophage cells comprising the few remaining inflammatory lesions (AR, 1000× magnification).

Lung sections from GKO mice were studied for the location of the bacilli after *M. tuberculosis* infection. The bacilli were visualized in lung sections via a modified staining method combining the auramine O and rhodamine B staining (AR) for acid fast bacteria, hematoxylin QS for staining tissue and DAPI for staining nuclei. At 18 days post aerosol infection, which is the start of treatment of our standard laboratory protocol for this model [43], the lung tissue sections mainly contained inflammatory lesions consisting primarily of macrophage (mΦ) and granulocyte (GC) cell accumulations arranged in an unorganized manner (Fig. 2A), as also earlier described [46]–[48]. Auramine-rhodamine positive (AR+) bacilli were found uniformly distributed across these inflammatory lesions (estimated at about 85–90%) (Fig. 2B), whereas a minority was found in and around airways, blood vessels and randomly dispersed throughout non-inflamed lung parenchyma. AR+ bacilli were predominantly intracellular during this early stage of infection within various mono- and multinucleated mΦ cell types (Fig. 2C, D). At a later stage (20 to 22 days post aerosol infection), there was a loss of alveolar septal architecture due to progressive inflammation and individual cell and lesion necrosis. It is at this stage that the AR+ bacillary population changes from being exclusively intracellular to becoming increasingly extracellular within the areas of necrosis. By 28 days post aerosol infection, which is about one week before mice would succumb to disease without effective drug intervention, multiple lesions coalesce and exhibit extensive lesion necrosis (Fig. 2E, F). Necrotic foci contained high numbers of both intracellular bacteria [within mΦ and multinucleated giant cells (55%)] (Fig. 2G) as well as extracellular AR+ bacilli [residing in alveolar spaces filled with necrotic cellular debris (40%)] (Fig. 2H). Few AR+ bacilli (5%) were visible in and around blood vessels and non-granulomatous lung parenchyma (Fig. 2I).

In the GKO mouse model, drug treatments start 14 to 18 days post aerosol exposure of *M. tuberculosis* and lasts for 9 to 14 consecutive days. After 4 days of drug treatment with INH or RIF in the GKO mice, there was a minimal effect observed on lesion histology as well as on the distribution of almost exclusively intracellular bacilli in the lesions. After 7 days of treatment, an initial reduction in lesion size and of AR+ bacilli number became evident. After 10 days of drug treatment, there was minimal inflammation remaining in the lung pathology. At this time, a reduction in AR+ bacilli was clearly evident and this reduction was seen uniformly across the entire tissue section. The AR+ bacilli were mainly located either within the few remaining inflammatory lesions or were situated peripherally within the adjacent alveolar spaces (Fig. 2J). A minority of AR+ bacilli was dispersed in non-inflamed lung parenchyma (5–10%). The remaining bacilli were primarily intracellular, which is as expected as treatment was initiated prior to the onset of necrosis (Fig. 2K, L).

### Progression of necrosis and development of hypoxia in late stage GKO

The necrotic granulomas found in the later stages of *M. tuberculosis* infected GKO mice showed similar morphology to necrotic, hypoxic granulomas observed in *M. tuberculosis* infected guinea pigs [22], [23], [26].

**Figure 3:**
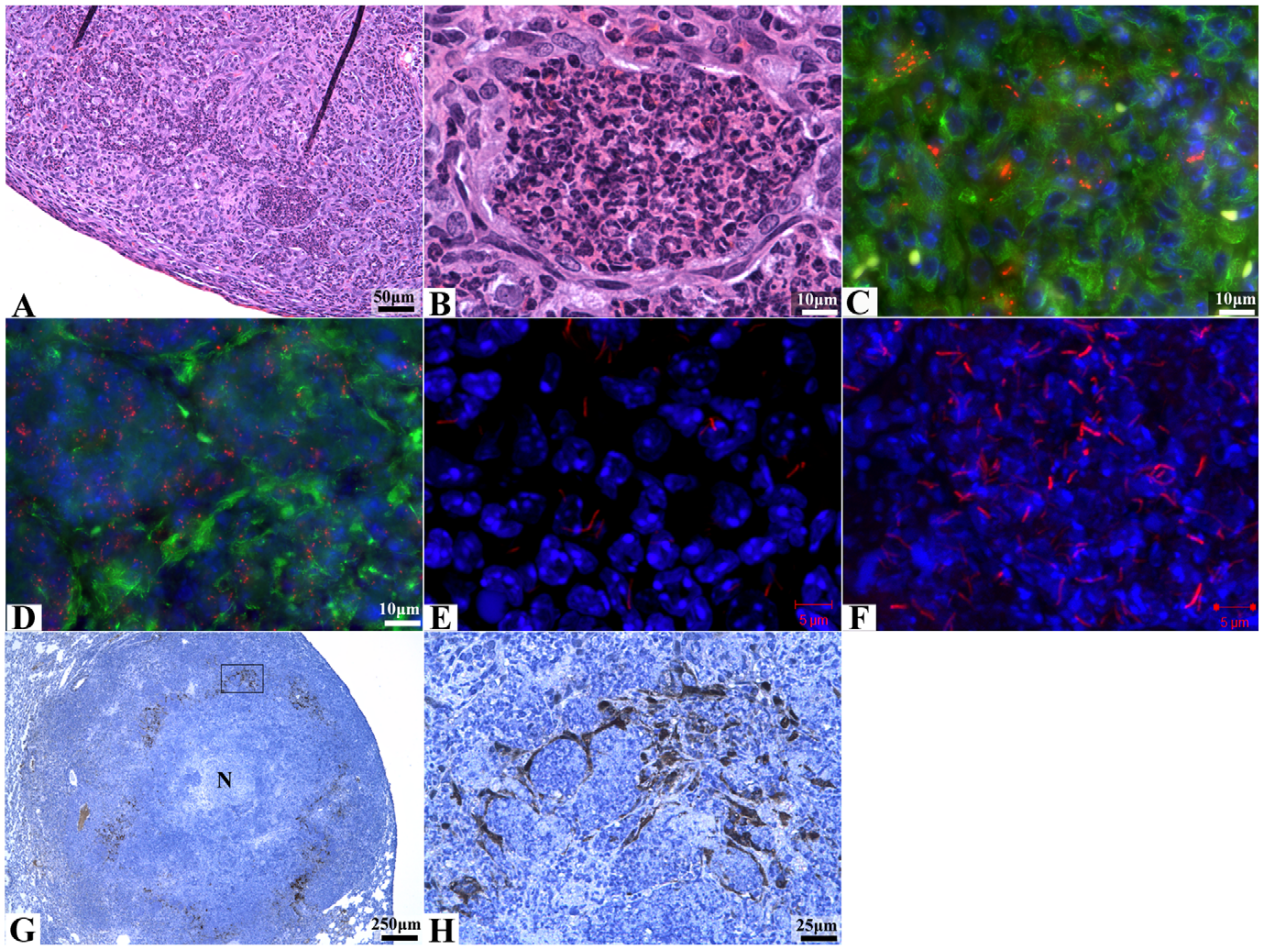
The progression of cellular necrosis that occurs within pulmonary inflammatory lesions during *M. tuberculosis* infection in GKO mice and the development of hypoxia as revealed by pimonidazole. (AR) = auramine-rhodamine, hematoxylin QS and DAPI; (H&E) = hematoxylin and eosin. (A) An inflammatory lesion from the lungs of *M. tuberculosis* infected GKO mice 22 days after aerosol infection. The lesion shows a number of alveolar spaces beginning to accumulate granulocytes prior to the presence of cellular necrosis (H&E staining, 200× magnification). (B) High magnification of the area demarcated in figure 3A (circle) showing an alveolus completely occluded with inflammatory infiltrate (H&E staining, 1000× magnification). (C) Fluorescent image of an inflammatory lesion from the lungs of *M. tuberculosis* infected GKO mice 18 days post-aerosol infection showing a highly organized tubulin network with intact nuclei (Immunofluorescence for Tubulin (green) and MTB (red) and DAPI (blue). 1000× magnification). (D) Immunofluorescent image of an inflammatory lesion from the lungs of *M. tuberculosis* infected GKO mice 28 days post-aerosol TB (red) infection showing loss of tubulin (green) architecture and degenerated nuclei (blue) indicating loss of cell viability (1000× magnification). (E) Confocal microscopy of an inflammatory lesion from the lungs of *M. tuberculosis* infected GKO mice 18 days post-aerosol infection. The nuclei (blue) appear healthy with few numbers of AR+ bacilli (red). (AR staining combined with DAPI; 630× magnification with additional 2.5× digital zoom). (F) Confocal microscopy of an inflammatory lesion from the lungs of *M. tuberculosis* infected GKO mice 28 days post-aerosol infection. Degenerated fragments of inflammatory cell nuclei are within alveoli with a high number of extracellular AR+ bacilli (AR staining combined with DAPI; 630× magnification with additional 2.5× digital zoom). (G) Immunohistochemistry detecting hypoxia (brown) in a necrotic lung lesion from an *M. tuberculosis* infected GKO mouse 29 days after aerosol infection. The area of central necrosis (N) is surrounded by epithelioid macrophages near a major airway. (Hematoxylin counterstain, 40× magnification). (H) High magnification of the area demarcated within the inflammatory lesion shown in figure 3G (square). The center of alveoli, which is likely hypoxic, is filled with cellular debris and fails to stain due to the lack of viable cells (Hematoxylin counterstain, 400× magnification).

Staining with hematoxylin and eosin showed a distinct increase in cellular necrosis over time. Early pathology (around 22 days post-infection) shows the aggregation of predominantly granulocytes within alveolar spaces (Fig. 3A, B). These cellular accumulations then begin to degenerate as observed by the progression from intact cells to necrotic, cellular debris (Fig. 2E, F). The accumulation of nuclear material within the alveolar spaces lead to an increase in basophilic staining during the progression of disease.

Confocal microscopy was utilized to confirm whether the bacteria were intra- or extracellular in the eukaryotic cells. Immunohistochemistry using anti-tubulin antibodies visualized the cytoskeleton, in combination with DAPI staining which stained the nuclei. The integrity or degeneration of the viable eukaryotic cells was thereby shown by the intactness of the nuclei via the DAPI staining, as well as by the organization of the tubulin structure via immunohistochemistry. Since every section is 5 microns only, the bacilli observed in a microscopy section will be in the same plain of focus as the viable cells, and the proximity of the bacillary location to the intact nuclei indicates their intracellular nature. At 18 days post infection, intact nuclei and highly organized tubulin network can be observed (Fig. 3C), whereas at day 28 the nuclei are ill-defined and the cytoskeleton structure is lost (Fig. 3D) indicating loss of cell viability. Confocal microscopy confirmed this observation by showing the transition from healthy appearing nuclei at day 18 (Fig. 3E) to accumulated nuclear debris within alveoli at day 28 in GKO mice (Fig. 3F).

To determine whether lesions in the lungs of GKO mice infected with *M. tuberculosis* are hypoxic, mice were injected i.p. with the hypoxia marker 2-nitroimidazole 1.5 h prior to euthanasia, as we previously described for the guinea pig model [23]. Mice were injected 15, 17, 20, 22, 25, and 29 days after aerosol. Hypoxia was not detected by pimonidazole staining in the lungs of *M. tuberculosis* infected GKO mice until 25 days post aerosol infection (data not shown). By 29 days, pimonidazole was observed to markedly stain around areas of necrosis in pulmonary lesions of the GKO mice (Fig. 3G, H). Hypoxia was not detected within areas of necrosis due to the absence of viable cells for the pimonidazole to form adducts with. These results indicate that the decreased oxygen conditions associated with tuberculosis disease in humans and other animal models are present in the lungs of GKO mice.

### Intracellular *M. tuberculosis* in C57BL/6 lungs

**Figure 4:**
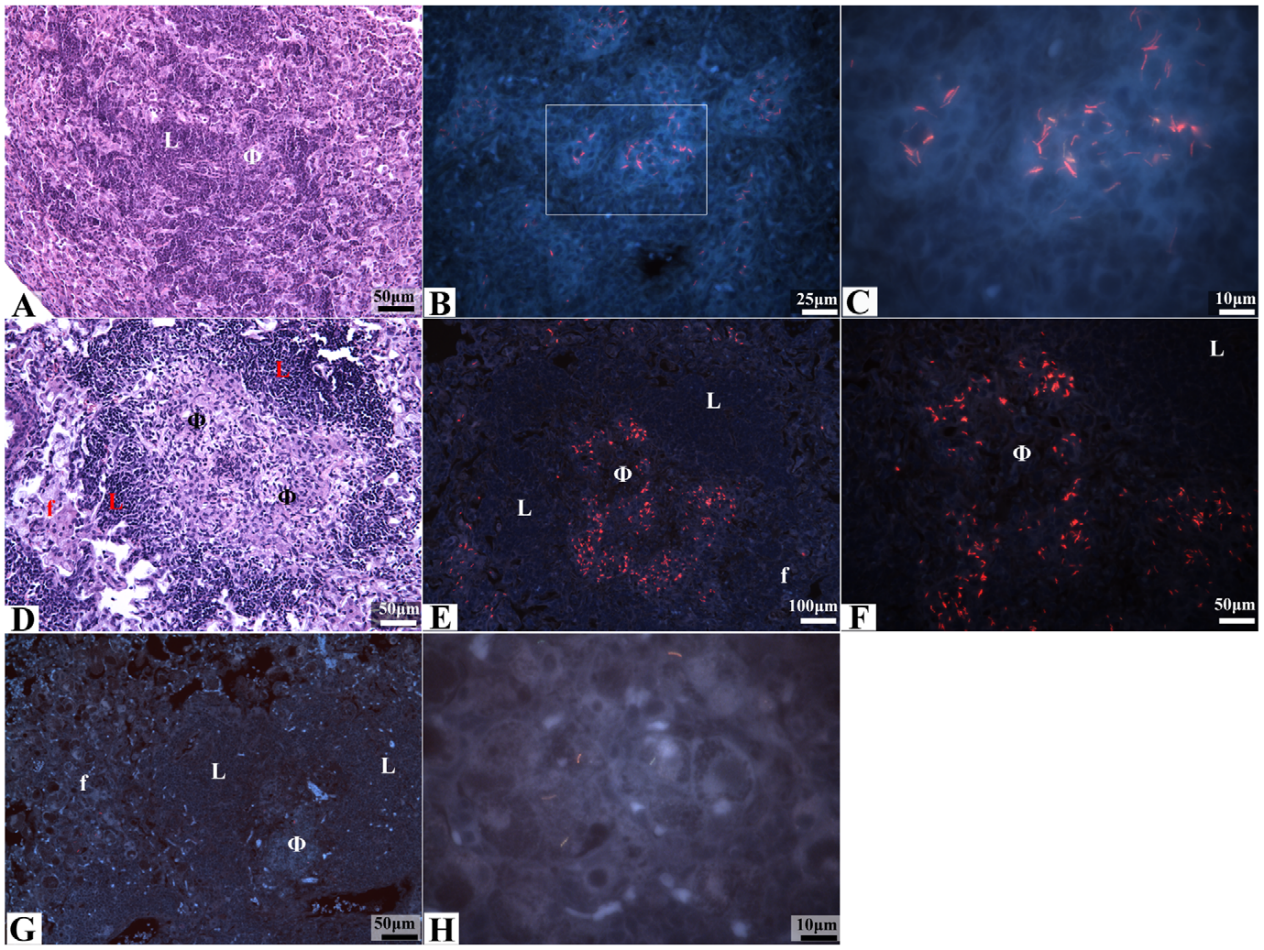
Intracellular *M. tuberculosis* bacilli in lungs from *M. tuberculosis* infected C57BL/6 mice, throughout infection and after 6 weeks of MXF drug treatment. (AR) auramine-rhodamine, hematoxylin QS and DAPI; (H&E) hematoxylin and eosin. (A) An inflammatory lesion from the lungs of an *M. tuberculosis* infected C57BL/6 mouse 4 weeks after aerosol infection. The cellular architecture of lesions shows a field of lymphocytes (L) surrounding multiple macrophage aggregates (Φ) (H&E staining, 200× magnification). (B) An inflammatory lesion from lungs of an *M. tuberculosis* infected C57BL/6 mouse 4 weeks after aerosol infection. Intracellular AR+ bacilli were predominantly found within macrophage rich regions surrounded by lymphocytes (AR, 400× magnification). (C) High magnification of the inflammatory lesion shown in figure 4B (circle) showing intracellular AR+ stained bacilli within macrophages (AR, 1000× magnification). (D) An inflammatory lesion from the lungs of an *M. tuberculosis* infected C57BL/6 mouse 9 weeks after aerosol infection. The lesion shows a distinct rim of lymphocytes (L) surrounding a core of epithelioid macrophages (Φ). A layer of foamy macrophages (f) can be seen surrounding the lymphocyte rim (H&E staining, 200× magnification). (E & F) Fluorescent images of low (E) and high (F) magnifications of the inflammatory lesion depicted in figure 4D taken from a serial tissue section. The majority of AR+ bacilli at this time are found in epitheloid mΦs (Φ) located within the lymphocyte cuff (L), whereas a lower number of AR+ bacilli are located in foamy mΦs (f) located at the peripheral edges of inflammation (AR, 100× and 200× magnifications). (G) Inflammatory lesion from the lungs of a C57Bl/6 mouse infected with *M. tuberculosis* and treated with moxifloxacin for 6 weeks. Cellular architecture of lesions after 6 weeks of drug treatment is similar to untreated controls and consist of a mΦ core (Φ) surrounded by lymphocytes (L) and foamy mΦs (f) (AR, 200× magnification). (H) High magnification of the peripheral edges of the inflammatory lesion shown in figure 4G. A few remaining AR+ bacilli are found within foamy macrophages located outside of the lymphocyte cuff (AR, 1000× magnification).

The location of bacilli was studied in lung sections from C57BL/6 mice after *M. tuberculosis* infection via a modified staining method combining AR for staining acid fast bacteria, hematoxylin QS for staining tissue and DAPI for staining nuclei. Four weeks after *M. tuberculosis* aerosol exposure, which is the start of drug treatment in our standard laboratory protocol for this mouse model [44], the inflammatory lung lesions consisted of large lymphocyte (LC) aggregates surrounding multiple, smaller accumulations of epithelioid mΦs (Fig. 4A) as previously reported [18], [49]. AR+ bacilli (∼95%) were found intracellular in the multiple epithelioid mΦ aggregates located within the lymphocyte field (Fig. 4B, 4C). A small proportion of AR+ bacilli (∼5%) were observed in foamy mΦs located adjacent to areas of inflammation. At nine weeks post aerosol infection, most of the inflammatory lesions displayed a well-defined, highly organized lesion structure that consisted primarily of a central region of epithelioid mФs and neutrophils surrounded by a distinct, sometimes incomplete, lymphocyte rim (Fig. 4D) as previously reported [18], [49]. These lesions sometimes exhibited a mild degree of tissue necrosis characterized by numerous, small foci of eosinophilia that were devoid of cellular debris. A distinct increase in AR+ bacilli number was observed at 9 weeks post aerosol infection, AR+ bacilli (80–85%) were found intracellular within mФs distributed throughout the central region of inflammatory lesions within the LC cuff (Fig. 4E, 4F). Fewer AR+ bacilli (15–20%) were found within foamy mФs that were located outside of the lymphocyte cuff in the surrounding alveolar air spaces. AR+ bacilli were not found in the areas of necrosis.

The location of *M. tuberculosis* in the lungs of C57BL/6 mice was studied after 6 weeks of INH, RIF or MXF treatment. Over the 6 weeks treatment, the bacterial burden determined by plating on agar plates decreased in the lungs from 6.00±0.07 Logs in the untreated control groups to 2.08±0.16, 3.06±0.38 and 1.75±0.07 Log10CFU for INH, RIF and MXF, respectively. The occurrence of drug resistant colonies after treatment is not an issue in this model due to the low bacterial load at the start of treatment. After two weeks of treatment, minimal effect was observed on lesion pathology and distribution of the intracellular AR+ bacilli across the pulmonary lesions. After six weeks of therapy the pulmonary inflammation was markedly reduced, although the same cell morphology, spatial distribution and degree of necrosis was still observed in remaining lesions. Despite limited effect on lung lesion pathology, a clear decrease in the number of AR+ bacilli in lungs was evident at this time and this reduction was uniform across the lesion. The bacilli (80–85%) were located intracellularly in epithelioid mФs within the lymphocyte cuff composing the lesion cores (Fig. 4G), while the minority (15–20%) were found outside of the lymphocyte cuff in foamy mФs (Fig. 4H). Remarkably, these AR+ bacilli could still be visualized in these foamy mФs after drug treatment. Although AR+ bacilli were often found in close proximity to necrotic regions, none were found within these necrotic sites.

### Extracellular *M. tuberculosis* in the primary lung lesions in guinea pigs

**Figure 5:**
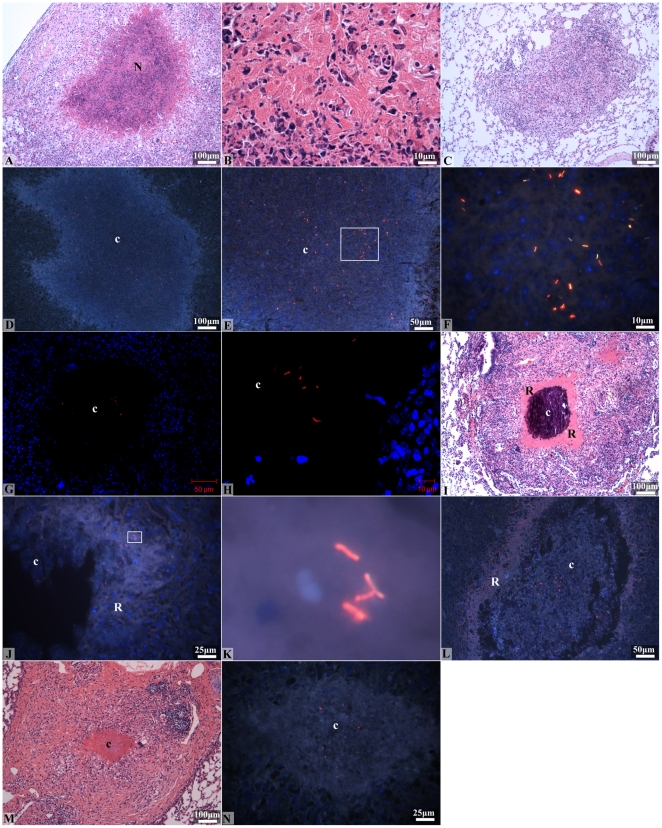
Location of *M. tuberculosis* bacilli in lungs from *M. tuberculosis* infected guinea pigs, throughout infection and after 6 weeks of INH or TMC207 drug treatment. (AR) auramine-rhodamine, hematoxylin QS and DAPI; (H&E) hematoxylin and eosin. (A, B) Low (A) and high (B) magnifications of an early primary granuloma in the lungs of *M. tuberculosis* infected guinea pigs 4 weeks post-aerosol infection. Primary granulomas are distinguished from secondary lesions by the presence of necrosis (N) (H&E staining, 100× and 1000× magnifications). (C) A secondary lesion in the lungs of an *M. tuberculosis* infected guinea pig 4 weeks post-aerosol infection. Secondary lesions are distinguished from primary granulomas by the lack of central necrosis in the former (H&E staining, 100× magnification). (D, E) Low (D) and high (E) magnifications of a primary granuloma from the lungs of an *M. tuberculosis* infected guinea pig 4 weeks after aerosol infection. The majority of AR+ bacilli are extracellular within the necrotic core (c) (AR, 100× and 200× magnifications). (F) High magnification of area demarcated in figure 5E (square). Extracellular AR+ bacilli in the necrotic core of primary granulomas exist as single cells or are situated in clusters (AR, 1000× magnification). (G, H) Confocal micrographs of a necrotic granuloma showing an acellular necrotic core (c) with extracellular AR+ bacilli (red) (AR and DAPI, 200× and 630× magnifications). (I) A primary granuloma from the lungs of an *M. tuberculosis* infected guinea pig 10 weeks after aerosol infection showing advanced calcification and calcification of the necrotic core (c) and the acellular rim (R) surrounding the core (H&E staining, 100× magnification). (J) Fluorescent image of a primary lung granuloma from an *M. tuberculosis* infected guinea pig 10 weeks after aerosol infection. Extracellular AR+ bacilli are present within the necrotic core (c) and the acellular rim (R) surrounding the necrotic regions (AR, 200× magnification). (K) Cropped image from figure 5I (square) showing extracellular AR+ stained bacilli in the acellular rim. (L) Fluorescent image of a necrotic primary lung granuloma from an *M. tuberculosis* infected guinea pig treated for 6 weeks with INH. Extra-cellular AR+ bacilli are primarily within the core (c) of the partially calcified lytic necrosis, and to a lesser extent within the acellular, uncalcified rim (R) (AR, 200× and 400× magnifications). (M) A low magnification of the remnant of a primary lung granuloma in an *M. tuberculosis* infected guinea pig treated for 6 weeks with TMC207. The primary granuloma shows a caseous necrotic core (c) surrounded by inflammatory cells (H&E staining, 100× magnification). (N) Fluorescent image of the caseous necrotic core (c) in the primary lung granuloma shown in figure 5M taken from a serial tissue section. The few extra-cellular AR+ bacilli remaining after TMC207 treatment are primarily located within the central core of caseous necrosis (AR, 400× magnification).

The location of bacilli in guinea pig lung sections was studied by using the combination staining of AR, hematoxylin QS and DAPI starting one month after low dose aerosol infection (which is the start of drug treatment in our standard laboratory protocol for the guinea pig model) [23], [45], [50]. Lung sections from guinea pigs studied four weeks after aerosol infection contained multiple granulomatous lesions with differing morphologies. The classic necrotic granulomas (also primary granuloma) were differentiated from inflammatory lesions (also secondary granuloma) based on the presence of necrosis surrounded by lymphocytic cell populations [22], [23], [25], [49], [51]–[53] (Fig. 5A, C). The AR+ bacilli (∼70%) were dispersed predominantly throughout the necrotic areas of the necrotic granulomas (Fig. 5D, E). The bacilli are present extracellularly either as small clusters of bacteria or as single cells (Fig. 5F). Confocal microscopy was then used to confirm the location of AR+ bacilli within the acellular necrotic core of these granulomas (Fig. 5G, H). Fewer AR+ bacilli (∼20%) were observed intracellularly within mФs found in close proximity to necrosis within primary lesions, and very few AR+ bacilli (∼10%) were found within mΦ cells from secondary lesions.

By 10 weeks post aerosol infection, the necrotic core of the primary granulomas progressed to a state of complete, dystrophic calcification [22], [52] (Fig. 5I). A modest decrease in AR+ bacilli was observed, but in contrast to earlier time points, the AR+ bacilli (95%) were now almost exclusively extracellular within the necrotic core and surrounding, acellular rim of residual lesion necrosis in the primary granulomas (Fig. 5J, K). Most secondary lesions contained very few intracellular, if any, detectable AR+ bacilli (data not shown).

Over the 6 weeks treatment, the bacterial burden determined by plating on agar plates decreased in the lungs from 5.12±0.28 Log10 CFU in the untreated control group to 3.76±0.0.1 and 1.78±0.26 Log10CFU for INH and TMC207, respectively. The occurrence of drug resistant colonies is not an issue in this model due to the low bacterial load at the start of treatment. INH treatment had a differential effect on the lung pathology of the primary versus the secondary lesions. Secondary lesions diminished in size and were largely healed after 6 weeks of INH treatment as previously reported [23]. Primary granulomas, on the other hand, required prolonged therapy in order to see visible resolution of the pathology. Healed secondary lesions within the pulmonary parenchyma reatain normal alveolar structure except for thickened alveolar walls and a mild to moderate increase in cellularity. Healed primary lesions are characteried by residual foci of dystrophic calcification and fibrosis that replace alvoli or are ebedded in the fibrous stroma that supports airways and pulmonary lymphatic and blood vascuature [53]. The AR+ bacterial population in the primary lesions steadily declined throughout INH treatment. However, the decrease in AR+ bacilli was far less pronounced in guinea pigs compared to the mouse infection models treated with the same drugs. Similar to the untreated controls, the remaining AR+ bacilli (∼95%) were extracellular located in primary granulomas and concentrated within the necrotic core and acellular rim, with only few intracellular bacilli in the surrounding mФ and lymphocyte region (Fig. 5L). Very few bacteria (∼5%) were found within mΦ cell types composing secondary lesions.

Besides INH treatment, we evaluated the highly potent TMC207 in the guinea pig model to see if more intensive treatment would ultimately result in visible resolution of the more advanced necrotic lesions. Drug efficacy data of TMC207 and INH in the guinea pig model have been reported before [23], and the drug treatments were repeated for this study also using intermittent timepoints of 2, 4 and 6 weeks of treatment. Similar to INH treatment, 6 weeks of TMC207 treatment lead to an almost complete clearance of secondary lesions in guinea pigs. However, compared to INH, TMC207 was more effective at resolving some of the primary granulomas in guinea pigs when administered for 6 weeks. While the numbers of AR+ bacilli were far lower after TMC207 treatment than after INH treatment, the remaining AR+ bacilli were predominantly present in the necrotic cores and surrounding acellular rims of the remaining primary granulomas (Fig. 5M, N).

## Discussion

TB drug discovery and development use a sequence of *in vitro* assays and animal models of *M. tuberculosis* infection to identify and select active experimental compounds. Mouse models provide important information regarding pharmacokinetics and *in vivo* efficacy of novel compounds [15]–[17], [54]–[57]. Recently, other animal models which reflect the progression of lung pathology as seen in humans (to include necrosis, calcification and fibrosis of lesions) are being considered in later stages of drug development: such as the guinea pig [21], [26], [53], [58]–[61], the rabbit [21], [28], the non-human primate infection model for tuberculosis [27], the Kramnik mouse [62], [63], and more recently the minipig model [64]. Surprisingly, there is limited to no information on the location of *M. tuberculosis* in the most widely used animal models for TB drug development. Up to date, the *in vivo* efficacy of a new compound is primarily measured by the reduction in bacillary load determined after plating of the organ homogenates on solid agar with little knowledge relating to pathology, location of the bacilli in the various animal models, or the potential differential drug activity in various locations in the lung. In this manuscript, we provide a comprehensive microscopic and histopathologic analysis of the disease progression of the animal models used in-house for TB drug development with a main focus on the location of the bacilli in the lung.

*M. tuberculosis* is generally thought of as being an ‘intracellular organism’ living in (and perhaps needing) its host cell, the macrophage. We show that this is not necessarily the case. *M. tuberculosis* bacilli were visualized via a new rapid staining method combining fluorescent acid-fast AR for staining bacteria, hematoxylin QS for staining tissue and DAPI for staining nuclei. This fluorescent staining method enabled us to easily visualize the bacteria in the tissue across a whole lung granuloma, whereas in past studies using the Ziehl-Neelsen method by brightfield microscopy, only limited information per microscopic field could be collected using high magnification. Confocal microscopy then gave us a three dimensional view of *M. tuberculosis* within a granuloma. Our earlier work using standard Ziehl-Neelsen staining suggested that the remaining bacilli after drug treatment in the guinea pig model were primarily present just inside the zone of incomplete necrosis within the acellular, fibrotic rim of necrotic lung lesions [23]. However, with this improved staining and detection method, the rim proved to be merely one of the locations. In this comprehensive study, we demonstrate that the majority of bacteria in the guinea pig model are more central within the core area of the necrotic lesion. In the mouse models studied here, we found that the majority of bacilli were indeed intracellular within macrophages through most of the infection. In the immunocompetent C57BL6 mice, AR+ bacilli were clearly intracellular, and this was especially evident in the foamy macrophages located outside of the lymphocyte region as described earlier [65], [66], [67]. In the immunocompromised GKO model, considerably more bacilli per macrophage were visualized by the AR staining method. Interestingly, in the late stages of infection in *M. tuberculosis* infected GKO mice, the granulomatous lesions begin to exhibit significant necrosis with the bacilli progressively becoming extracellular (with about half of the bacilli eventually being extracellular). The GKO mice form massive granulomas that contain multiple focal-points of necrotic cellular debris accumulating in alveolar air spaces. These necrotic areas increase in number and size and in a late stage can collectively coalesce into a single core (which is also observed in guinea pig, as well as in human lesions). As a confirmation of necrosis in the late stage GKO mouse model, immunofluorescence for tubulin was used that showed a clear loss of the cytoskeleton structure of the eucaryotic cells. The presence of macrophages and debris in the airways in this late stage of the GKO model shows pathological similarity to lung inflammation in cavitary TB patients. Since an earlier study using the guinea pig model showed these necrotic lesions to be hypoxic using pimonidazole [23], this led us to investigate the oxygen tension in lungs of *M. tuberculosis* infected GKO mice. Hypoxia has been postulated as one of the environmental conditions which transitions mycobacteria *in vitro* into a non-replicating phase, thereby affecting their responsiveness to drugs [7], [68]. In the late stage of the GKO model, a clear hypoxic zone was found at the periphery of the necrotic regions. The inability of pimonidazole to stain the necrotic tissue itself was due to the lack of viable cells which are required to enzymatically create detectable adducts, however this necrotic tissue is also likely to be hypoxic.

The guinea pig model was studied, which shows more complex pathology with a heterogeneity of lesion types and stages within one animal at any one time. The location of bacilli in guinea pig lung lesions was also studied by fluorescent AR staining. Intracellular bacilli were present in inflammatory lesions only in the first weeks after aerosol infection showing equal numbers of intra- and extracellular bacilli visualized throughout the granuloma. From one month after low dose aerosol infection, the bacilli were mainly extracellular within the necrotic core and surrounding acellular rim of the primary granulomas. The ‘core of the lesion’ is here referred to as the centre of necrotic guinea pig lesion that calcifies and mineralizes, whereas the ‘rim of the lesion’ is defined here as the edge of incomplete necrosis which has not yet calcifieded. Confocal microscopy clearly showed that the core of the granuloma is indeed acellular and devoid of intact nuclei and is the location where most bacteria are. The few intracellular bacilli found were either located in the outer lymphocyte and epithelioid macrophage cell layer of primary granulomas or in secondary lesions, although this latter location rarely contained any detectable AR+ *M. tuberculosis* bacilli at all. The reason for finding only very few acid-fast bacilli in the secondary granulomas is not entirely clear. The secondary lesions in the guinea pig model are thought to originate mainly from bacilli after hematogenous dissemination from the primary lesions, which occurs only after the immune response is already activated. Therefore, bacillary replication in the secondary lesions is likely curtailed due to the now activated immune response [51]. The difference in cytokine profiles in primary and secondary lesions has been described earlier by others with secondary lesions showing a strong anti-inflammatory response [69]. For TB drug development, it is important to realize that in case of progressive disease most bacteria are primarily extracellular organisms within a matrix of cellular debris and calcified cellular components. TB drug treatment aims to sterilize lesions from bacteria and should therefore target to eradicate these initial necrotic lesions from the extracellular bacteria.

Interestingly, a diffuse pink staining effect was frequently seen after AR staining in primary necrotic granulomas of the guinea pigs after day 30 (figure 5L). This pink haze was evident throughout the necrotic granuloma and was also seen in the GKO mouse in areas of advanced necrosis at day 28 post infection (figure 2H) but not earlier, and was never observed in the standard C57BL/6 mouse. It is not entirely clear what this diffuse staining represents, however, it is possible that AR staining detects free mycolic acids either from degraded dead bacilli or from bacilli that shed mycolic acids, a feature reported for *in vitro* pellicle-grown *M. tuberculosis* [70]. We have shown free mycolic acids to stain readily with rhodamine but not auramine in our laboratory (data not shown) in an ongoing study to elucidate the potential target of the AR stain.

Once the vast differences in bacillary locations in the different animal models were recorded, we studied the effect of drug treatment on the clearance of bacilli in these different locations in the lungs using the new fluorescent acid-fast AR stain combined with hematoxylin QS and DAPI. Results showed that for both mouse models the reduction of the AR+ bacilli after drug treatment was largely homogenous across the lesion. Necrosis was never seen in the lungs of the drug-treated GKO mice as the onset of necrosis only occurs late in infection after treatment has initiated. In the unorganized lesion structure of the GKO mice, a very rapid clearance of AR+ bacilli was observed, presumably by the large number of phagocytic cells and macrophages present. Therefore, the AR staining method can in fact be used for the GKO model as a readout for drug efficacy. In immunocompetent mice, clearance of the AR+ bacilli occurs slower . After drug treatment, the clearance of bacilli is homogenous across the lesions; the remaining bacilli are intracellular, either in epithelioid mФs within the LC cuff or in foamy mФs located outside the LC cuff. Although difficult to quantify, a slight trend was observed of relatively more bacilli remaining in the foamy mФs versus the epithelioid mФs after drug treatment. Whether this is due to slower clearance of dead bacilli by the various macrophage celltypes, or to the bacilli in the foamy mФs being less responsive to drugs has to be further investigated. Interestingly, the foamy mФs have more recently been described as being the key participants in both sustaining persistent bacteria and contributing to tissue pathology that leads to cavitation and the release of infectious bacilli [65]–[67], [71], [72]. Cardona et al. hypothesize this foamy macrophage population to be dynamic and drain from the alveolar spaces resulting in dissemination and generating intragranulomatous necrosis [72].

In the guinea pig model of *M. tuberculosis* infection, drug treatment reduced the bacterial load in the lungs heterogeneously over the different lung lesion types. While drug treatment completely reversed lung inflammation associated with secondary lesions relatively quickly, the resolution of the primary granulomas required more intensive treatment regimens [45], [53]. These results are in keeping with classical studies by Smith *et al.*, who showed that INH, RIF and PZA chemotherapy sterilized secondary lesions but had far less effect on the primary lesions [73]. None of the earlier studies, however, described the location of the bacilli in the lung. We observed that six weeks of INH treatment of *M. tuberculosis* guinea pigs only had a moderate effect on the AR+ bacilli visualized mainly present in the primary granulomas, whereas in the guinea pigs treated with TMC207 for 6 weeks an almost complete eradication of AR+ bacteria in both lesion types was observed. The clearance of the bacteria in the necrotic lesions is generally very slow, and therefore AR staining may not be a good alternative to CFU enumeration for evaluating drug efficacy in guinea pigs, unless the bacteria are lysed after drug treatment (which is in fact observed after TMC207 treatment *in vitro*, personal communication, K. Andries). The few remaining AR+ bacilli after TMC207 treatment were found extracellular, in the core of the primary lesion, a microenvironment of residual primary lesion necrosis. These observations further highlight the importance of a TB drug to eradicate the persisting, extracellular bacteria remaining within the necrotic lesions.

Although acid-fast stains have been used for diagnostic purposes for decades, the exact cellular component of *M. tuberculosis* recognized by the dyes is still being debated. Fuchsin, the main component of Ziehl-Neelsen and Kinyoun acid-fast stain, has been shown to stain the vastly complex lipid portion of the mycobacterial cell wall. However, less is known about the target of the combined auramine O-rhodamine B stain. Auramine O is believed to bind to mycolic acids and nucleic acids [74]–[76]. Although rhodamine B has been used in numerous *M. tuberculosis* studies, the exact staining target of *M. tuberculosis* has yet to be elucidated [74]. Preliminary studies in our laboratory show that rhodamine B alone stains *M. tuberculosis* quite readily in culture and tissue sections and also stains purified cell wall components (data not shown). To understand what bacterial population is visualized with the AR staining method in this study, we need to keep two characteristics of the stain in mind. First, the AR stain might not visualize all bacterial populations present in the lesion, we might not see the earlier described acid-fast negative bacterial subpopulation(s) [77], [78]. Recently, we also reported on an acid-fast-negative but immunofluorescence-positive *M. tuberculosis* population *in vitro* as well as *in vivo* [12]. Secondly, the stain cannot differentiate between live and recently killed, intact [and not yet cleared] tubercle bacilli. Despite these limitations, the purpose of the combined staining method was to illustrate the differences in locations of the AR+ bacilli and their clearance (or lysis) caused by drug treatment, throughout entire lung sections across our animal models.

To understand the interaction between immunology, pathology and the location and state of the *M. tuberculosis* bacillus, the role of the immune response was studied by comparing single drug treatment effects in the immunocompromised GKO mice versus the immunocompetent C57BL/6 mice. The immune response is generally believed to be favorable to the host by containing and aiding in the eradication of bacilli due to the induction of protective cytokines and other immune molecules [6], [79]. From the perspective of TB therapeutics however, the immune response appears to be an unfavorable response in that it renders the bacilli less responsive to drugs, as is shown by the results described here. Drug activity was found to be far more pronounced for all compounds evaluated in the GKO mice when compared to the immunocompetent C57BL/6 mice. Drugs administered were INH, which is mainly active against replicating bacilli [68], as well as RIF and/or quinolones (MXF, GATI), which are known to be effective against replicating bacteria as well as sterilizing compounds [80], [81]. This result is not entirely surprising because the bacilli are actively replicating in the GKO mice without the interference of adaptive immunity of the host. In fact, *M. tuberculosis* bacteria in GKO mice replicate about once a day, which is the same replication rate as *in vitro* cultures. However, drugs showed far less activity in the C57BL/6 mice with any given compound when compared to the GKO mice. Earlier papers have elegantly shown that bacteria are slowing their replication rate in immunocompetent mice due to the immune response and show a changed metabolism in the chronic disease state in mice [11], [36], [82]. The results show an advantage of the GKO model as a first *in vivo* screen to assess potential *in vivo* activity. The high numbers of actively replicating bacilli in GKO mice make for a large dynamic window when testing novel experimental compounds for *in vivo* activity.

The findings presented in this paper have at least three important implications for TB drug development. First, it is important in TB drug development to realize that the majority of the bacilli in an advanced disease state are extracellular organisms located in necrotic lesions, as is also seen in human cavitary disease [83]. Most mouse models are modeling activity of single compounds or drug regimens against intracellular bacteria, and because of this limitation other animal models with a more progressive pathology are required to evaluate drug efficacy against potentially persistent, extracellular bacilli in hypoxic, necrotic lesions. A second implication is that a decrease in inflammation might not be a good measure of antibactericidal activity. Recently, different imaging methods have been introduced as methods to follow the effect of TB drug therapy in real time such as PETscan (which requires the uptake of radio-labeled glucose by actively metabolizing cells) and CATscan based imaging modalities [21], [63]. Our results show that rapid resolution of non-necrotic inflammatory lesions (such as the secondary lesion in the guinea pig model) might not adequately reflect the bactericidal activity of a drug as these lesions only contain very few bacilli. And thirdly, the immune response as well as the rapid structural organization of the tuberculous granuloma is usually seen as a favorable host response as it contains bacilli locally, thus preventing the progression of the disease. However, the immune response and its effect on lung pathology may decrease the drug responsiveness of the bacilli. In the context of drug therapy, the necrotic granuloma may in addition also present a physical barrier to effective treatment and to the host immune response. Therapies aiming at preventing and minimizing necrosis may be beneficial in eliminating bacilli that persist in necrotic lesions in the face of standard TB therapy.

In summary, the differences in host immune response among the different animal models infected with *M. tuberculosis* result in a wide variety of granulomatous lesion types which will eventually determine efficacy of a TB drug regimen. Increasing our understanding on lesion morphologies as well as the location of bacilli among the different animal models is vital to designing different levels of stringency for testing new drugs.

## Materials and Methods

### Ethics Statement

All experimental protocols were approved with written consent by the Animal Care Use Committee of Colorado State University (approval numbers ACUC # 04-302A-06 and ACUC # 06-221A-03) which abides by the USDA Animal Welfare Act and the Public Health Service Policy on Humane Care and Use of Laboratory Animals.

### Bacterial Isolates

The virulent *M. tuberculosis* Erdman strain (TMC 107; ATCC 35801) has been used as a standard for drug evaluations in mice in our laboratory, and the strain was propagated as previously described [14], [44]. Briefly, *M. tuberculosis* Erdman was grown to mid-log phase in Proskauer-Beck Medium containing 0.05% Tween 80 (Sigma Chemical Co., St. Louis, MO) and stored in vials frozen at −70°C until use.

The strain *M. tuberculosis* H37Rv (Trudeau Institute, Saranac Lake, NY) has been used as a standard in our laboratory for guinea pig infection studies [23], [84]. *M. tuberculosis* H37Rv was grown from low passage seed lots in Proskauer-Beck liquid medium containing 0.05% Tween 80 to early mid-log phase and frozen in aliquots at −70°C until needed.

### Chemicals and drugs

Isoniazid (INH), rifampin (RIF) and ethambutol (EMB) were obtained from Sigma Chemical Co. (St. Louis, MO). Moxifloxacin (MXF) and gatifloxacin (GAT) were kindly provided by Southern Research Institute (SRI) (Birmingham, AL). All drugs, except for RIF, were dissolved in water. RIF was dissolved in 100% dimethyl sulfoxide (DMSO) prior to dilution in distilled water (5% final DMSO concentration). Drug formulations prepared in distilled water were prepared weekly and stored at 4°C. For the guinea pig studies, INH was dissolved in 40% (wt/vol) sucrose and administered in 1 ml per guinea pig. INH was prepared weekly in distilled sucrose water and stored at 4°C. TMC207 was kindly provided by Dr. K. Andries (Tibotec, Belgium) and was prepared in a hydroxypropyl-ß-cyclodextrin solution (CD) as described before [23].

### Immunocompromised mouse TB infection model

Several experiments using different lengths of treatment were performed,. Briefly, eight- to ten-week-old female specific pathogen- free, C57BL/6-Ifngtm1ts (GKO) mice were purchased (Jackson Laboratories, Bar Harbor, Maine). These mice have a disrupted interferon-γ gene, which renders them highly susceptible to tuberculosis [47]. The standard infection and treatment protocol for this model was performed as extensively validated and previously described [43]. Mice were exposed to a low-dose aerosol infection with the *M. tuberculosis* strain Erdman ( TMC 107; ATCC 35801) in a Glas-Col inhalation exposure system (Glas-Col Inc., Terre Haute, IN) [14]. One day after low dose aerosol infection, three mice were euthanized to verify bacterial uptake of 50 to 100 CFU per mouse. Each treatment group consisted of 4–5 mice for every subsequent time point. Treatment was initiated 18 days after low dose aerosol infection (standard protocol) and lasted up to 28 days after aerosol infection. Untreated mice cannot succumb to disease 28–30 days after aerosol infection. INH was administered at 25 mg/kg, GAT and MXF at 100 mg/kg, and all drugs were administered via oral gavage for 7 days/week. One control group of untreated, infected mice was euthanized by CO_2_ inhalation at the start of treatment, and a second control group after the cessation of treatment. Mice were euthanized after 2, 5, 7, and 10, days after the start of treatment.

### Immunocompetent mouse TB infection model

Several experiments using different lengths of treatment were performed and the protocols are described below. Six- to 8-week-old female specific pathogen-free immunocompetent C57BL/6 mice (Charles River, Wilmington, MA) were infected via a low dose aerosol exposure to *M. tuberculosis* Erdman. The standard infection and treatment protocol for this model were performed as previously described [14], [44], [85]. Three mice were euthanized one day post low dose aerosol to verify bacterial uptake of 50 to 100 CFU per mouse.

For the short term treatment experiment, drug treatment started three weeks after low dose aerosol infection (standard protocol) and lasted for 7 days. INH was administered at 25 mg/kg, EMB at 150 mg/kg, MXF at 100 mg/kg and RIF at 10 mg/kg, and drugs were administered via oral gavage for 7 days. Groups of 5 mice were euthanized after 0, 2, 5 and 7 days of drug treatment.

For the long term treatment experiment, drug treatment was started 3 weeks post-infection and continued for 12 weeks. Five infected mice were euthanized at the start of treatment as pretreatment controls. INH was administered at 25 mg/kg and RIF at 20 mg/kg, 5 days per week via oral gavage. Mice were euthanized at 2, 6, and 12 weeks after the start of treatment.

### Guinea pig TB infection model

Four to five month-old, female Hartley guinea pigs (Charles River, Wilmington, MA) weighing approximately 500 g each were exposed to a low-dose aerosol of *M. tuberculosis* in a Madison aerosol chamber device. Our standard infection and treatment protocol for guinea pigs was followed as previously described [50], [86]. Guinea pigs were delivered a low inoculum resulting in approximately 20–30 lesions in the lungs. At 30 days post-infection, 5 guinea pigs were euthanized to determine the bacterial load at the start of treatment. Each guinea pig was drug treated by administering each dose in the back of the mouth (5 guinea pigs per group). Control groups received daily oral administration of 1 ml of 40% (wt/vol) sucrose. Drug treated groups were administered INH at 30 mg/kg for 5 days per week, or TMC207 at 15 mg/kg. Earlier pharmacokinetic data using validated HPLC assays yielded an AUC 0–24 of 18.22 hr*mg/ml for INH at 30 mg/kg [45]. Our earlier *in vivo* efficacy data on TMC207 in the guinea pig model showed the 15 mg/kg dose as a safe and highly efficacious dose [23].

### Bacterial enumeration on agar plates

Mice were euthanized by CO_2_ inhalation, and left lung lobes were aseptically removed and disrupted in a tissue homogenizer as previously described [44]. The number of viable organisms was determined by plating serial dilutions on nutrient Middlebrook 7H11 agar plates (GIBCO BRL, Gaithersburg, MD) containing cycloheximide at 10 µg/mL and carbenicillin at 50 µg/mL. Guinea pigs were euthanized by sodium barbital injection (Sleepaway; Fort Dodge Laboratories), and organs were aseptically removed and plated as previously described [23]. Statistical analysis to assess the significance of treatment efficacy results on bacterial numbers remaining after drug treatment was performed for mouse models [44] and guinea pig models [23], as described before.

### Combined staining procedure to visualize bacteria with AR and surrounding tissue

The caudal right lung lobe for mice and guinea pigs was infused *in situ* with 10% neutral-buffered formalin. Four micron thick paraffin sections were stained with TB Auramine-Rhodamine T as recommended by Becton-Dickinson (Sparks, MD), with modifications in order to visualize bacteria in the surrounding lung tissue. A combination staining method was developed by using both auramine O and rhodamine B to detect acid fast bacteria (AR; Becton Dickinson), hematoxylin QS for staining tissue (HQS; Vector Laboratories, Inc., Burlingame, CA) and 4′6-diamidino-2-phenylindole dihydrochloride for staining nuclei (DAPI; Sigma Chemical Co., St. Louis, MO). Tissue sections were dewaxed in xylene and rehydrated through a graded alcohol series, then stained with TB Auramine-Rhodamine T for 30 min. After washing excess stain with ddH_2_O, slides were decolorized with TB Decolorizer TM (BD) until the stain appeared dissolved. Counterstaining was performed with hematoxylin QS for about 5 sec. After washing excess hematoxylin with ddH_2_O, slides were stained for 15 min. with DAPI (200 ng/ml final concentration) and washed in ddH_2_O.

Entire lung tissue sections were scored based on: 1) estimating the number of auramine-rhodamine positive (AR+) bacilli per tissue section in mice and accurately counting the AR+ bacilli in guinea pig tissues (numbers are presented as percentages), 2) bacillary appearance (individual or clustered), and 3) intra or extra-lesional presence. For mouse lung tissues, bacterial numbers were generated from 2 non-sequential lung lobe sections for every timepoint for five mice per treatment group. Scoring sheets were generated for every lung section with the number of lesions showing bacilli for every section and the approximate number of the bacilli per lesion for the C57BL/6 mice (tables not shown). For the GKO mouse model, the percentages of bacteria present per lesion versus the whole lesion was estimated due to the high bacillary burden. For guinea pig tissues, the actual bacterial numbers were counted from 2 non-sequential lung lobe sections from three guinea pigs from every treatment group (tables not shown), for the primary (necrotic) and secondary (inflammatory) lesions. The improvement of the pathology after drug treatment in the guinea pig lung was evaluated blindly by a veterinary pathologist by scoring the lung involvement of the inflammation, as described earlier [24], [45].

### Immunofluorescence

Five micron sections of lung tissue were dewaxed in xylene and rehydrated through graded alcohols. Antigen retrieval was performed using the Retriever™ 2100 which pressure cooks at 121°C for 15 minutes using Target Retrieval Buffer solution S3307 (DAKO, Carpinteria, California). Blocking was performed with 1% goat serum in PBS (Biomeda, Foster City, California) for 30 minutes. The slides were incubated at 4°C overnight with a monoclonal mouse anti-tubulin antibody (Cell Signaling Technology, Danvers, Massachusetts) and a rabbit polyclonal anti-TB whole cell lysate minus LAM (Antibody E293, CSU TB Vaccine Testing and Research Materials Contract, Colorado). Subsequently, the slides were washed using PBS and the antibody was detected with an Alexafluor 488 labeled goat anti-mouse IgG and also an Alexafluor 568 labeled goat anti-rabbit IgG (Invitrogen, Carlsbad, California). The slides were washed in PBS, mounted with ProLong® Gold antifade reagent with DAPI (Invitrogen, Carlsbad, California) and photographed under a fluorescent microscope.

### Hypoxia staining with pimonidazole

Pimonidazole staining was used as described before [19], [23] to detect hypoxic regions in the lungs of GKO mice. Pimonidazole is a 2-nitroimidazole that is able to identify regions of hypoxia (<4 µM O_2_ saturation or O_2_ tensions of 10 mm Hg) in animal organs after injection. Pimonidazole forms protein adducts with thiol groups in cells adjacent to hypoxic regions and the adducts are detected with a monoclonal antibody. *M. tuberculosis*-infected GKO mice were injected intraperitoneally (i.p.) with pimonidazole hydrochloride (Chemicon, Hampshire, United Kingdom) at a dose of 60 mg/kg mouse body weight dissolved in 1× PBS at 1.5 h prior to sacrifice at days 15, 17, 20, 22, 25, and 29 after low dose aerosol infection. Five micron thick sections of paraformaldehyde-fixed, paraffin-embedded tissue were cut and mounted on slides for processing. Tissue sections were deparaffinized with xylene before performing antigen retrieval with pronase (Fisher Scientific, Schwerte, Germany) for 40 min. at 40°C. Endogenous peroxidase activity was reduced with 1% hydrogen peroxide in TBS for 20 min. at room temperature (RT) in the dark. The blocking reagents avidin D and biotin were then added to each slide for 15 min. each. Sections were blocked with a mouse-on-mouse Ig blocking reagent (Vector Laboratories, Inc., Burlingame, California) for 1 hour at RT. Biotinylated antimouse IgG was pre-labeled with the anti-pimonidazole antibody for 10 min. and was applied to slides overnight at 4°C. Liquid DAB (DAKO, Carpinteria, California) was applied to slides for 5–10 min. to visualize the reaction. Finally, slides were counterstained with Gill's Hematoxylin (Sigma Chemical Co., St. Louis, MO) for 10 min. and cover-slipped.

### Fluorescent and Confocal Microscopy

Photographs were taken on a Nikon Eclipse 80i with DAPI, FITC and TRITC filter sets and an Optronics Microfire color fluorescent camera. Multiple photographs were taken under different focal planes when necessary and combined using the Extended Depth of Focus (EDF) function on Nikon NIS Elements AR 3.0 software. Confocal images were captured on a Zeiss LSM 510 Meta laser scanning confocal microscope and analyzed using Zeiss LSM image analyzer version 4.0.
